# Improving implementation of smoking cessation guidelines in pregnancy care: development of an intervention to address system, maternity service leader and clinician factors

**DOI:** 10.1186/s43058-021-00235-5

**Published:** 2021-11-17

**Authors:** Megan E. Passey, Catherine Adams, Christine Paul, Lou Atkins, Jo M. Longman

**Affiliations:** 1 The University of Sydneys, University Centre for Rural Health, PO Box 3074, Lismore, NSW 2480 Australia; 2 Northern New South Wales Local Health District, Locked Mail Bag 11, Lismore, NSW 2480 Australia; 3 School of Medicine and Public Health, University of Newcastle, Callaghan, NSW 2308 Australia; 4 UCL Centre for Behaviour Change, University College London, London, WC1N 3AZ UK

**Keywords:** Behaviour Change Wheel, Theoretical Domains Framework, Stakeholder engagement, Smoking in pregnancy, Smoking cessation, Antenatal care

## Abstract

**Background:**

Smoking during pregnancy increases the risk of multiple serious adverse infant, child and maternal outcomes, yet nearly 10% of Australian women still smoke during pregnancy. Despite evidence-based guidelines that recommend routine and repeated smoking cessation support (SCS) for all pregnant women, the provision of recommended SCS remains poor. Guidance on developing complex interventions to improve health care recommends drawing on existing theories, reviewing evidence, undertaking primary data collection, attending to future real-world implementation and designing and refining interventions using iterative cycles with stakeholder input throughout. Here, we describe using the Behaviour Change Wheel (BCW) and the Theoretical Domains Framework to apply these principles in developing an intervention to improve the provision of SCS in Australian maternity services.

**Methods:**

Working closely with key stakeholders in the New South Wales (NSW) health system, we applied the steps of the BCW method then undertook a small feasibility study in one service to further refine the intervention. Stakeholders were engaged in multiple ways—as a core research team member, through a project Advisory Group, targeted meetings with policymakers, a large workshop to review potential components and the feasibility study.

**Results:**

Barriers to and enablers of providing SCS were identified in five of six components described in the BCW method (psychological capability, physical opportunity, social opportunity and reflective and automatic motivation). These were mapped to intervention types and we selected education, training, enablement, environmental restructuring, persuasion, incentivisation and modelling as suitable in our context. Through application of the APEASE criteria (Affordability, Practicability, Effectiveness, Acceptability, Side effects and Equity) in the stakeholder workshop, behaviour change techniques were selected and applied in developing the intervention which includes systems, clinician and leadership elements. The feasibility study confirmed the feasibility and acceptability of the midwifery component and the need to further strengthen the leadership component.

**Conclusions:**

Using the BCW method combined with strong stakeholder engagement from inception resulted in transparent development of the MOHMQuit intervention, which targets identified barriers to and enablers of the provision of SCS and is developed specifically for the context in which it will be implemented. The intervention is being trialled in eight public maternity services in NSW.

**Supplementary Information:**

The online version contains supplementary material available at 10.1186/s43058-021-00235-5.

Contributions to the literature

- Recommendations for developing complex interventions include drawing on existing theories, reviewing published evidence, undertaking primary data collection, being attentive to future implementation in real-world settings and designing and refining interventions using iterative cycles with stakeholder input throughout the process.
- We report an exemplar of this process, with the development of an intervention using the Behaviour Change Wheel (BCW) and Theoretical Domains Framework (TDF), with strong engagement from stakeholders at multiple levels of the health system, repeatedly throughout the process—many other interventions have been developed with limited stakeholder input.
- We demonstrate how the careful use of the BCW and TDF allowed identification of critical environmental barriers to provision of smoking cessation support (SCS), including lack of leadership for SCS and inadequate systems to support clinicians to provide SCS. While others have developed training programmes for antenatal clinicians to improve their provision of SCS, we are not aware of any that have explicitly addressed leadership for SCS nor the broader systemic issues such as the capability of the EMR to support clinicians in this role.
- We also demonstrate how the inclusion of a small feasibility study in the development process identified both strengths and weaknesses of the intervention, supporting further refinement of the intervention prior to a larger trial.

## Background

Smoking during pregnancy increases the risk of multiple serious adverse infant outcomes including stillbirth, preterm birth, low birth weight, asthma and childhood respiratory infections [1–5]. In 2018, 9.2% of pregnant women in Australia smoked in the first half of pregnancy, and of these, 80% smoked in the second half of pregnancy [6]. While antenatal smoking has declined in recent years, this decline has been unequal and disparities persist: with a persistently high prevalence of smoking among Aboriginal and Torres Strait Islander women (43%), teenage mothers (31%), those living in remote (18%) and very remote (36%) areas, and those of low socio-economic status (17%) [6].

While many pregnant women are highly motivated to quit [7] and are interested in receiving care to achieve this [8–10], they often face substantial challenges including a lack of effective support from clinicians [11]. Evidence from systematic reviews indicates that psychosocial interventions to support pregnant women to quit are effective [1, 12]. The missing link, however, is a failure to consistently implement effective cessation care for pregnant women.

Evidence-based Australian guidelines [13] developed by NSW Health recommend routine, repeated smoking cessation support (SCS) for all pregnant women using brief interventions based on the 5As (*Ask, Advise, Assess, Assist and Arrange follow-up*) and include providing nicotine replacement therapy (NRT) if women are otherwise unable to quit. The 5As are effective and considered best practice, with wide adoption internationally [14–17]. However, provision of recommended SCS to pregnant women has remained persistently poor both in Australia and elsewhere [10, 18–21]. A state-wide survey of women’s experiences of maternity care in NSW found that only 46% of women who smoked in pregnancy recalled being told about quitting programmes [22]. Our own research confirmed midwives, obstetricians and managers all reported major gaps in care, particularly in assisting women with cessation strategies and arranging follow-up [23, 24], both of which are crucial to quitting success [25, 26].

**Fig. 1:**
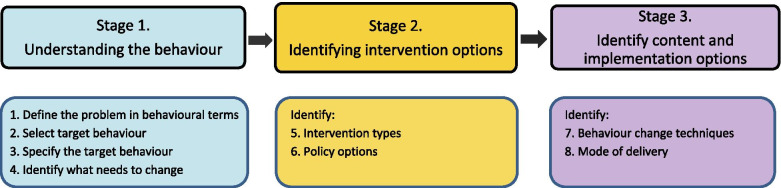
Behaviour change intervention design method

There is strong evidence that changing the behaviour of health care providers with persistently poor practice requires the use of a comprehensive evidence-based and theory-driven approach which addresses specific behaviours in specific contexts [12, 27]. One approach, the ‘Behaviour Change Wheel’ (BCW) [28] was developed from 19 frameworks of behaviour change and is based on the COM-B model of behaviour where Capability, Opportunity and Motivation are all required to enact a particular behaviour. The BCW presents a structured approach to developing interventions, with three stages, within which are eight steps—see Fig. 1. We selected the BCW for the development of our intervention as this method starts with the behaviour, allows for a comprehensive analysis of barriers to and enablers of the behaviour and offers a systematic way of selecting intervention content to target barriers and enablers. Furthermore, the clear specification of behaviour change techniques supports subsequent evaluation of implementation fidelity, testing mechanisms of action and future replication.

The aim of this study was to use the BCW to develop an intervention to improve implementation of the smoking cessation in pregnancy guidelines [13] in public antenatal care, in NSW, Australia. In Australia, 75% of births occur in public hospitals, and 94% of women have at least five antenatal care visits [6]. While several different models of care are available (e.g. midwifery led, obstetric led or GP shared-care), all women intending to birth in a public hospital should have received at least one antenatal visit within the public health system, and most will have received the majority of their antenatal care in the public system. Thus, improving SCS in these settings has the potential to have a significant impact on the wellbeing of women and their babies. While it would also be beneficial to improve SCS among GPs and obstetricians working in private practice, addressing the barriers in these settings would likely require very different approaches, and thus they are not specifically included in this intervention.

The project was initiated from an ongoing collaboration between academic researchers and a senior Clinical Midwifery Consultant (CMC) (CA) due to evidence of suboptimal implementation of SCS recommendations in Australian maternity services [10, 18]. In NSW, the Ministry of Health determines overall policy and strategy, with clinical care provided by 15 Local Health Districts (LHDs). Throughout the process, we involved stakeholders at multiple levels of the NSW Health system and from several disciplines, which helped strengthen the engagement of these key partners and our understanding of the context in which the intervention would operate. Here, we report the process followed, the intervention developed and the outcomes of a small feasibility study of the intervention.

## Methods

The multidisciplinary research team consisted of a public health physician (MP), a social scientist (JL), two researchers with expertise in behaviour change and implementation science (CP&LA), and a senior midwife (CA). All had prior experience in smoking cessation research. At the start of the project, we met with senior officials in NSW Health (covering tobacco control, midwifery and obstetric care) and successfully secured their support for the project and its implementation. The project was guided throughout by an Advisory Group consisting of clinicians (midwives and obstetricians), maternity care managers, policymakers and tobacco control experts, including those involved in the Aboriginal Maternal Infant Health Service and those recommended at the initial meetings with NSW Health. The Advisory Group provided advice and direction to the project, assisted with implementation and ensured relevance and consistency with state policies and initiatives. At several points, the Advisory Group specifically engaged us with relevant state developments to optimise the project outcomes and opportunities for translation into policy and practice. These included contributing to changes to the electronic medical record (EMR) system for maternity services and assisting in the development of new smoking cessation training modules for clinicians.

We followed the stages and steps described in the BCW book [28] and shown above (Fig. 1), then undertook a small study in one site to assess the feasibility of implementing the intervention and acceptability of its components. These steps are detailed below, and the intervention developed (Midwives and Obstetricians Helping Mothers to Quit (MOHMQuit)) is described at the end of the ‘Results’ section at step 8, with subsequent revisions based on the feasibility study described just prior to the ‘Discussion’ section.

### Stage 1. Understanding the behaviour

#### Step 1. Define the problem in behavioural terms

We used Australian smoking cessation guidelines for use in pregnancy [13] and documented evidence-practice gaps identified in the literature [10, 18, 19, 22] to define the problem and the target population.

#### Step 2. Select target behaviour

As the guidelines recommend provision of the 5As at every antenatal visit, we accepted this as the target behaviour and did not generate any alternatives.

#### Step 3. Specify the target behaviour

Based on the guidelines and our knowledge of the NSW Health maternity care system, the research team specified who, where, when, how often and with whom the target behaviours should occur, in consultation with the Advisory Group.

#### Step 4. Identify what needs to change

We undertook two related studies, using the Theoretical Domains Framework (TDF) to inform data collection and analysis [29]. The TDF is a validated comprehensive framework for identifying barriers and enablers to behaviour change and includes 14 theoretical domains that can be mapped to the six components of the COM-B model (physical capability, psychological capability, physical opportunity, social opportunity, reflective motivation and automatic motivation) [28]. We undertook an initial qualitative study with 27 maternity service managers, obstetricians and midwives working in the public health system in NSW, using the TDF to guide the interviews in order to identify the barriers and enablers to provision of SCS [23]. We then conducted an online anonymous survey with midwives working in public antenatal clinics in NSW, with the questionnaire based on the TDF, and refined using findings from the qualitative study [24]. As these studies are already published, we have not presented detailed methods here. Data from both these studies were mapped to the TDF domains and the COM-B components by MP and JL, with review and discussion with the remaining authors. Through this process, we identified the barriers that needed to be addressed to support behaviour change as well as enablers to assist.

### Stage 2. Identify intervention options

#### Step 5. Identify intervention types

Using the published BCW guidance, we mapped the identified TDF domains and COM-B components to the full range of possible intervention types [28]. These potential intervention types were then reviewed by the research team against the APEASE criteria (Affordability, Practicability, Effectiveness and cost effectiveness, Acceptability, Side effects/safety and Equity), considering relevance and feasibility within the context of the NSW public antenatal care system, with reduction in the number retained.

#### Step 6. Identify policy options

Provision of SCS as a core part of antenatal care is already informed by NSW Health guidelines [13], required by NSW Health policy, and is an identified NSW Health priority with training in smoking cessation available to midwives. Additionally, a new electronic medical record system (*e*Maternity) was in development and the research team was contributing to enhancements in the section on smoking, as a result of our initial findings, in parallel with progressing our own intervention development. Consequently, no further policy assessment was undertaken.

### Stage 3. Identify content and implementation options

#### Step 7. Identify behaviour change techniques

The intervention types identified during step 5 were mapped to a potential long list of behaviour change techniques (BCTs) using the BCT Taxonomy v1, which includes 93 different BCTs, and is provided in the BCW book [28]. These were discussed by the research team and an initial shortlist developed based on our knowledge of the context in which we were working. We then reviewed the BCTs using the APEASE criteria. Following this initial review, the research team developed a ‘prototype intervention’ which specified the intervention types and BCTs in detail.

The ‘prototype intervention’ was then presented to a stakeholder workshop including members of the Advisory Group and additional policymakers, midwives (including Aboriginal midwives), midwifery managers, educators, smoking cessation specialists and trainers and researchers. The workshop participants (*n* = 24) worked in groups to review the intervention types and BCTs using the APEASE criteria, recommending inclusion, modification or exclusion. Following this, further discussions were held with senior policymakers in NSW Health, who provided advice on their preferences regarding fine-tuning the intervention. Final decisions on the inclusion of intervention types and BCTs were then made by the research team.

#### Step 8. Identify the mode of delivery

The research team considered the options for delivery of the intervention within the context of the NSW Health system and developed a detailed implementation plan, including identification of resources required. Discussions with the Advisory Group members and notes from the earlier stakeholder workshop helped inform this process. Development of the resources included input from additional experts and stakeholders, including pregnant and postpartum smokers. The process of resource development is the subject of a forthcoming paper [30].

### Feasibility and pilot study

The final stage of development was a feasibility and pilot study at one site to assess operational feasibility and acceptability of the intervention and its components, among midwives and the maternity service leadership team. Assessing feasibility and acceptability prior to a large trial is recommended in order to identify any problems and confirm that the intervention can be delivered as intended [31, 32]. Due to resource constraints, this study did not include the component targeting obstetricians, although this component will be included in the larger trial. The aims of the pilot study were to assess the acceptability of the MOHMQuit intervention to maternity service leaders (managers, educators and those in other leadership roles) and midwives; the feasibility of implementing the leader and midwifery components of the MOHMQuit intervention; the extent to which participants used the various resources and processes developed; participants’ suggestions for changes or enhancements to the MOHMQuit intervention; participants’ perceptions of the value of implementing MOHMQuit more broadly, possible barriers and potential solutions; and the acceptability, face and content validity of data collection tools planned for use in a larger (subsequent) trial; in order to refine the intervention prior to a larger implementation trial. The approach was based on the theoretical framework of acceptability [33].

**Fig. 2:**
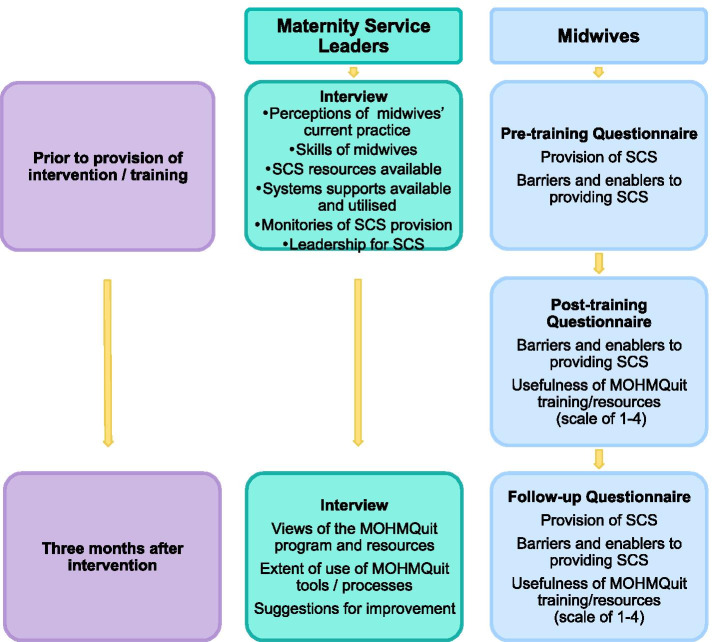
Data collection for the feasibility study

The study involved running both the leader and midwifery components and provision of all resources. Data collection is summarised in Fig. 2. Maternity service leaders were interviewed prior to the provision of the intervention and 3 months later.

Participating midwives completed questionnaires pre-training, post-training and 3 months later. Questionnaires were based on our validated questionnaire previously used in the state-wide survey of midwives to assess provision of SCS and barriers and enablers to doing so [24]. Twenty items in the original questionnaire relating to barriers/enablers were found to form three factors (‘Capability’, ‘Work environment’ (relates to opportunity in the COM-B) and ‘Personal priority’ (relates to motivation in COM-B)), which strongly predicted provision of SCS [24]. To assess the acceptability, face and content validity of the data collection tools, participants were also asked to provide feedback on relevance, acceptability and clarity of the questions; comprehensiveness of coverage of the issues; question order; time taken to complete; and any suggestions for improvement.

## Results

### Stage 1. Understand the behaviour

#### Step 1. Define the problem in behavioural terms

The problem identified was the gap between the guideline-recommended SCS and current practice identified in the literature, which was particularly acute regarding provision of assistance and arranging follow-up, rather than asking about smoking status [10, 18, 19, 22–24].

#### Step 2. Select the target behaviour

The target behaviour was delivery of the 5As (*Ask, Advise, Assess, Assist and Arrange follow-up*) at every antenatal visit as recommended in the guidelines, with the primary target being the *Assist* and *Arrange follow-up* elements, as these were most poorly provided [13]. For women who have recently quit, this same process is followed to identify and prevent relapse.

#### Step 3. Specify the target behaviour

We specified the target behaviour as midwives providing the 5As as specified in the guidelines, at every antenatal clinic appointment, with all pregnant women identified as smokers or who have quit smoking in the last 12 months. While both midwives and obstetricians provide antenatal care to women in the NSW public health system, all women are seen by midwives, and midwives have greater involvement in behavioural concerns than obstetricians, so we initially identified midwives as the target group for our intervention.

#### Step 4. Identify what needs to change

Barriers to provision of SCS were identified in five of the six components of the COM-B (psychological capability, physical opportunity, social opportunity and reflective and automatic motivation) and 10 of the 14 domains of the TDF, with changes required in each. We also identified enablers which could potentially assist with addressing the barriers, in four of the TDF domains and three of the COM-B components. These key themes and their corresponding COM-B components and TDF domains are shown in Table 1. Further detail is provided in Additional file 1.

**Table 1:** Barriers to and enablers of the provision of smoking cessation support, TDF domains and COM-B components, potential intervention types and BCTs initially identified

Theme	COM-B ***TDF domain***	Potential intervention types	BCTs
**Barriers**			
Clinician knowledge of the guidelines was poor and many were unaware that the 5As should be delivered at every visit [23, 24].	Psychological capability *Knowledge*	Education	Education • 4.1 Instruction on how to perform a behaviour (*information on how and when to provide the 5As*) • 6.3 Information about other’s approval (*explain Ministry of Health policy and guidelines*) • 9.1 Credible source (*above information provided by credible source*)
There was some confusion regarding the value of cutting down versus quitting [23].	Psychological capability *Knowledge*	Education	Education • 5.1 Information about health consequences (*provide information on the risks/benefits of quitting vs cutting down*)
Many clinicians reported poor knowledge and communication skills related to the Assist component, in particular (i) assisting motivated women with strategies to quit (including use of NRT), (ii) assisting to motivate women to try to quit who are not currently motivated and (iii) arranging follow-up [23, 24].	Psychological capability *Cognitive and interpersonal skills*	Training	Training • 4.1 Instruction on how to perform a behaviour (*detailed information on how to assist women with strategies including the use of NRT in pregnancy, and how to motivate them*) • 6.1 Demonstration of the behaviour (*demonstration of how to perform each of the elements of assisting under varying circumstances*) • 8.1 Behavioural practice/rehearsal (*practice performing the elements of assisting using role play*)
There were no mechanisms or systems for clinicians to use to monitor/self-monitor if they were following the 5As [23, 24].	Psychological capability *Behavioural regulation*	Education Enablement	Education • 2.2 Feedback on the behaviour (*provide information on clinic performance providing 5As*) Enablement • 1.2 Problem solving • 1.4 Action planning • 2.3 Self-monitoring (*encourage midwives to problem solve barriers and solutions to self-monitoring, and make a plan to manage this*)
Many clinicians thought that the 5As took too long to deliver within the context of a busy antenatal visit [23, 24].	Physical opportunity *Environmental context and resources*	Training	Training • 4.1 Instruction on how to perform a behaviour (*reiterating 5As designed to be delivered in a short consultation*) • 6.1 Demonstration of the behaviour (*how to have 5As discussions while doing other clinical duties*) • 8.1 Behavioural practice/rehearsal (*practice performing the elements of assisting using role play*)
There were no service-wide systems to identify smokers at subsequent visits and remind clinicians to deliver 5As [23, 24].	Physical opportunity *Environmental context and resources*	Environmental restructuring	Environmental restructuring • 7.1 Prompts/cues (*options: modify EMR to include flags for smokers (now complete), build in reminders to follow 5As at every antenatal visit and ensure key fields are included for all antenatal visits. If modifying EMR is not possible, develop a linked add-on decision support system; or develop paper-based reminders*) • 12.5 Adding objects to the environment (*as above for 7.1, and add posters and colourful, prominent lists in the clinic*)
There was no system to monitor smoking cessation support that women received, for quality assurance purposes [23, 24].	Physical opportunity *Environmental context and resources*	Training Environmental restructuring	Environmental restructuring • 12.5 Adding objects to the environment (*develop a reporting system for managers to monitor cessation support provided*) Training • 4.1 Instruction on how to perform a behaviour (*train key leaders in the use of the reporting system*)
Lack of resources, e.g. printed materials to use with women who smoke were unavailable, out of date or not specific to pregnancy [23, 24].	Physical opportunity *Environmental context and resources*	Environmental restructuring	Environmental restructuring • 12.5 Adding objects to the environment (*might be printed pamphlets, or links to online resources which can be centrally updated*)
Lack of leadership for smoking cessation and a lack of champions at all levels including both managers and peers [23, 24].	Social opportunity *Social influences*	Enablement	Enablement • 3.1 Social support (unspecified) (*buddy identified at training to provide ongoing support*) • 12.2 Restructuring the social environment (*manager encouraging attendance at training; discussion of 5As in meetings*) • 2.2 Feedback on behaviour (*develop mechanism to allow the manager to monitor progress on provision of 5As (or some component) and encourage this at team meetings/display in staff room, etc.*)
Some midwives lacked confidence to deliver the 5As, especially assisting women who were struggling [23, 24].	Reflective motivation *Beliefs about capabilities*	Persuasion Incentivisation Enablement	Persuasion • 15.3 Focus on past success and 15.1 verbal persuasion about capability (*highlight communication skills midwives have developed in other areas*) • 9.1 Credible source (*delivered by senior or other respected midwives*) Incentivisation • 10.4 Social reward (*praise for practising behaviour during and between intervention training sessions*) Enablement • 3.1 Social support (unspecified) (b*uddy identified at 1*^*st*^ *training to provide ongoing support*)
Some midwives did not consider referral to Quitline to be effective [24].	Reflective motivation *Beliefs about consequences*	Education Persuasion	Education • 5.1 Information about health consequences (*that referrals to Quitline result in x% increase in quit attempts/rates*) Persuasion • 9.1 Credible source (*delivered by Quitline staff or other cessation experts*)
Some midwives have concerns about damaging the client relationship [23, 24].	Reflective motivation *Beliefs about consequences*	Persuasion Modelling	Modelling • 6.1 Demonstration of the behaviour (*video showing engaged client and effective midwife*) Persuasion • 5.1 Information about health consequences and 5.3 information about social and environmental consequences and 6.3 information about others’ approval (*professional patient describing health and emotional (not valued) consequences of midwife not addressing their smoking—gives impression OK to keep smoking*) • 9.1 Credible source (*above information delivered by a pregnant or postpartum woman who smoked*)
Prioritising other health issues (e.g. gestational diabetes) over smoking [23].	Reflective motivation *Goals*	Persuasion	Persuasion • 5.1 Information about health consequences and 5.2 salience of consequences (*impact of smoking relative to another condition they manage well, e.g. diabetes*) • 9.3 Comparative imagining of future outcomes and 13.2 framing/reframing (*reframing action for smoking by comparing with action for other conditions*)
Framing smoking as a social issue/lifestyle choice rather than an addiction (and therefore not my role) [23].	Reflective motivation *PR&I*	Education Persuasion	Education • 5.1 Information about health consequences (*impact of nicotine on the brain and role in addiction*) Persuasion • 9.3 Comparative imagining of future outcomes (*comparison with their successful responses to other behavioural issues, e.g. domestic violence*) • 13.2 Framing/reframing (*reframing smoking as a behavioural indicator for intervention rather than a ‘lifestyle choice’*)
Some midwives are uncomfortable asking about smoking [23, 24].	Automatic motivation *Emotion*	Persuasion Modelling	Modelling • 6.1 Demonstration of the behaviour (*video showing engaged client and effective midwife*) Persuasion • 5.1 Information about health consequences and 5.4 information about social and environmental consequences (*professional patient describing health and emotional (not valued) consequences of midwife not addressing their smoking*) • 9.1 Credible source (*professional patient*) • 5.4 Information about social and environmental consequences (*other midwives feel good about professional role after delivering 5As*) • 9.1 Credible source (*midwifery champion*)
**Enablers**			**How these were used to support the intervention**
Knowledge of harms associated with smoking was reasonably good [23, 24].	Psychological capability *Knowledge*	Education Training Enablement	It was agreed not to address this specifically in our intervention as knowledge of harms was reasonably good and is also covered in the HETI modules, and there were more pressing issues to address
Midwives have good communication skills generally and are a trusted source of information for women [23].	Psychological capability *Cognitive and interpersonal skills*	Education Training Enablement	This enabler was fundamental to the overall intervention which leverages midwives’ excellent communication skills and strong trusting relationships with women to address smoking. It was specifically used in addressing the barriers related to lack of confidence to deliver the 5As
5As can be delivered while carrying out other clinical tasks [23].	Physical opportunity *Environmental context and resources*	Training	Some midwives had described how they provided the 5As while undertaking clinical tasks, so we developed a video to demonstrate this, and included a discussion of the ways in which this could be achieved in the workshops
The EMR prompts to ask about smoking at the initial visit [23].	Physical opportunity *Environmental context and resources*	Environmental restructuring	This was recognised as an important first step in identifying smokers and was considered in addressing the barrier related to the lack of systems to identify smokers at subsequent visits and reminding clinicians to deliver the 5As, and in the barrier regarding the lack of systems for monitoring for quality assurance purposes. We identified a number of options using ‘Environmental Restructuring’ to address this (see above and text) and subsequently contributed to enhancements to the EMR to address this.
Some clinicians reported increased role satisfaction from delivering 5As [23, 24].	Reflective motivation *Professional role and identity*	Persuasion	This was used to address the barrier that some midwives were uncomfortable asking about smoking, by developing a video resource with midwives talking about their satisfaction and the professional benefits of addressing smoking

#### Early translation activities

 In discussions of these findings with the Advisory Group, we were informed that NSW Health was implementing a new EMR for maternity services—*e*Maternity. The initial version was in the testing phase but still did not flag smokers and had not addressed the issues identified above. Through members of the Advisory Group, we worked with the developers to ensure some of these issues were addressed including automatic identification of women who smoke in a ‘banner’ on the screen along with any other ‘Considerations’ specific to that woman, making them easy to identify at subsequent visits; fields for recording smoking status and cigarettes/day at any visit; and spaces to record referrals and support provided. This process occurred in parallel with the intervention development.

### Stage 2. Identify intervention options

#### Step 5. Identify intervention types

The results of the research team’s assessment of each of the potential intervention types against the APEASE criteria are shown in Additional file 2. We selected Education, Training, Enablement, Environmental restructuring, Persuasion, Incentivisation and Modelling, as these all met at least half the APEASE criteria, with the remaining criteria unclear and requiring further consultation in step 7. We rejected Coercion and Restriction as neither of these met any of the APEASE criteria and were considered inappropriate in this context. The intervention types selected for each of the identified barriers are shown in column 3 of Table 1.

#### Step 6. Identify policy options

As explained in the methods, no further policy assessment was undertaken.

### Stage 3. Identify content and implementation options

#### Step 7. Identify behaviour change techniques

Initially, the research team shortlisted 22 different BCTs with several of these addressing multiple barriers. For example, BCT *5.1 Information on health consequences* was used to address the barrier ‘Prioritisation of other health issues over smoking’ by considering the impact of smoking relative to other conditions that are well managed (e.g. diabetes), and the barrier ‘Some midwives did not consider referral to Quitline to be effective’ by providing information that referrals to Quitline result in an increase in quit attempts/rates, as well as several other barriers. These are shown in column 4 of Table 1. The results of the review of the intervention types and corresponding BCTs by the stakeholder workshop are shown in Additional file 3. The majority of the BCTs were supported, with minimal change, and were retained. The workshop also recommended inclusion of Aboriginal health workers in the training programme for midwives as they play a key role in antenatal care for Aboriginal women. Following the workshop and discussions with senior NSW Health policymakers, decisions included [1] continue working with NSW Health in further refining *e*Maternity rather than developing a stand-alone system [2]; work with the NSW Health Education and Training Institute (HETI), which was developing a new online education module to improve clinicians support for smoking cessation in pregnancy—MP was invited to participate in the Expert Group advising HETI, and findings from step 4 above were used in designing the modules—these modules address some of the education and persuasion elements required [3]; inclusion of a component for the obstetric team, as the Ministry of Health emphasised that smoking cessation was core business for all clinicians, not just midwives.

#### Step 8. Identify the mode of delivery

The MOHMQuit intervention includes multiple components and modes of delivery (see Table 2). It was designed specifically to address gaps in existing programmes and meet the expressed concerns and needs of antenatal clinicians, at both individual and system levels, increasing the likelihood of uptake. All participants will be asked to complete the HETI online modules prior to participating in MOHMQuit, to ensure knowledge of harms of antenatal smoking, the evidence for SCS, use of the 5As, and use of NRT in pregnancy. These modules include the intervention functions Education, Persuasion and Modelling.

**Table 2:** The MOHMQuit intervention components and modes of delivery

Target group	Aims	Workshops	Resources
**Maternity service leadership group**: • Midwifery unit managers • Clinical midwifery consultants • Clinical midwifery educators • Other senior midwives	To enhance leadership for provision of SCS and achieve culture change by building: • Psychological capability • Physical opportunity • Social opportunity • Reflective motivation	3-h workshop covering: • SCS leadership • Reviewing *e*Maternity reports (local performance data on provision of SCS) • Action planning • Developing care pathways for SCS • Developing and maintaining champions	• Template for generating *e*Maternity reports on provision of SCS in their clinic • Guidance on the use of the *e*Maternity reports for quality improvement • Comparison with action for other conditions, e.g. gestational diabetes • A clinic/service action planning tool • Guidance on developing champions • Guidance on developing local care pathways
**Midwives and Aboriginal health workers:** • All midwives providing antenatal care in any setting • All Aboriginal health workers providing antenatal care in any setting • N.B. settings may include hospital or community-based clinics, outreach programmes and hospital wards	To increase provision of effective and appropriate SCS by enhancing: • Psychological capability • Physical opportunity • Reflective motivation	Full-day workshop covering: • Importance of providing SCS • How to provide effective SCS • Reviewing *e*Maternity reports (local performance data on provision of SCS) • How to use the MOHMQuit resources • Using tools for self-monitoring provision of SCS and action planning • Documentation of SCS in *e*Maternity	• 11 short videos demonstrating critical techniques in providing SCS • Guidance on recording smoking information in *e*Maternity • Information on NSW Quitline • Comparison with action for other conditions, e.g. gestational diabetes • Summary guide of the 5As • Assist and arrange follow-up flip booklet • Helpful hints for clinicians • Reference card to pin on badge holder • Self-help booklet for use with women • NRT information sheets for clinicians • NRT information for women
**Obstetricians and obstetric trainees involved in antenatal care**	To increase provision of effective and appropriate SCS by enhancing: • Psychological capability • Physical opportunity • Reflective motivation	3-h training covering: • Importance of providing SCS • How to provide effective SCS • Use of the MOHMQuit resources • Documentation of SCS in *e*Maternity	• 11 short videos demonstrating critical techniques in providing SCS • Guidance on recording smoking information in *e*Maternity • Information on NSW Quitline • Comparison with action for other conditions, e.g. gestational diabetes • Summary guide of the 5As • Assist and arrange follow-up flip booklet • Helpful hints for clinicians • Reference card to pin on badge holder • Self-help booklet for use with women • NRT information sheets for clinicians • NRT information for women

The *maternity service leadership* workshop will occur prior to the clinician-focused components to enhance leadership in supporting clinicians to provide SCS and will be conducted by a senior midwifery trainer. The workshop and accompanying resources include the intervention functions:

- Education: about the policy and guidelines to use the 5As and how and when to deliver them
- Training: how to provide leadership in smoking cessation support, how to identify and support champions and how to use the resources provided including running the *e*Maternity reports and using them for quality improvement (audit and feedback), using the clinic action planning tool and developing local care pathways for smoking cessation
- Environmental restructuring: providing a template for running *e*Maternity reports on provision of SCS in their clinic with guidance on how to use the reports for quality improvement (audit and feedback) and providing an action planning tool to use to audit policy and procedures in the clinic then use this for action planning
- Enablement: the importance of encouraging/supporting staff attendance at training and of encouraging regular discussions of SCS at team meetings, discussing mechanisms they could use to support team discussions and communicate progress (e.g. displays in the staff room) and brainstorming mechanisms and action planning to improve implementation of SCS
- Persuasion: providing information on the effectiveness of Quitline and the importance of referrals; comparing smoking with diabetes regarding the health consequences and current service provision, to clarify the extent of the gap in assisting smoking cessation; and reframing smoking as an addiction, rather than a lifestyle choice

On completion of the workshop, leaders will be asked to encourage and support clinical staff to attend the relevant workshops (see below), review *e*Maternity reports monthly and discuss with their team, complete the action planning tool and review annually, develop local care pathways for smokers and execute other actions guided by their action plan.

The workshops for *clinicians* (midwives, Aboriginal health workers, obstetricians and obstetric trainees) use evidence-based behaviour change techniques shown in Table 1 (e.g. social comparison, modelling, behavioural practice/rehearsal, reframing smoking) and will be jointly provided by a midwifery trainer and a smoking cessation trainer.

These workshops and resources use the following intervention functions:

- Education: about the policy and guidelines to use the 5As and how and when to deliver them
- Training: on how to provide the 5As effectively, including motivating change, providing assistance and follow-up, and integrating it into other clinical activities, using demonstration (videos), role play and feedback, and how to use the MOHMQuit resources
- Environmental restructuring: (in addition to the changes to *e*Maternity at the state level) guidance on recording smoking information in *e*Maternity; a range of resources to remind them to provide the 5As and how to do so (training videos, clinic posters, summary guide of the 5As, helpful hints for clinicians, assist and arrange follow-up flip booklet, a reference card for their badge holder, information on NRT); and resources to use with women (a self-help booklet and NRT information)
- Enablement: brainstorming ways to self-monitor provision of SCS and action planning to address this
- Persuasion: providing information on the effectiveness of Quitline and the importance of referrals; comparing smoking with diabetes regarding the health consequences and current service provision, to clarify the extent of the gap in assisting smoking cessation; reframing smoking as an addiction, rather than a lifestyle choice; highlighting the excellent communication skills that midwives already have that can be used to address smoking; and patient testimony on the importance of having cessation supported, and how it feels if it is not addressed
- Incentivisation: praise for providing SCS from leaders and champions
- Modelling: video demonstrating how to provide SCS without damaging the relationship

A 1-day workshop for midwives and Aboriginal health workers will be provided (run at least twice at each site to allow all midwives and Aboriginal health workers to attend), while a 3-h workshop will be provided for medical staff during regular clinical meetings. Relative to obstetric staff, the workshop for midwives and Aboriginal health workers will cover additional material and include role play, consistent with the greater role they usually play in behaviour change interventions. This structure and approach is consistent with usual practice regarding upskilling in the NSW Health system. Midwifery educators will provide training for new staff using a train-the-trainer model [34]. As an incentive for participation, continuing professional development points will be awarded.

#### Feasibility study results

The 3-h leader training was attended by three of four invited managers, but none of the three invited clinical educators, who had prior commitments. The full-day midwives’ training was attended by 12 of 15 midwives invited (27 were identified as eligible but 12 were not invited). All (leaders and midwives) had completed the HETI training in preparation for MOHMQuit.

Both the leaders’ training and the midwives’ training were well received, with positive feedback on the day and high scores on the midwives’ surveys (see Table 3). There were some minor suggestions for improvement of the programme at the training, mostly logistical. Additionally, in both the post-training survey and the follow-up survey, midwives requested more ongoing education or follow-up training. Following the training comments from midwives included: “I feel really motivated to do this because I now understand how important it is, I have skills and I’m confident to try it out” and “Amazing day, needs to be made mandatory for all midwives in the LHD”.

**Table 3:** Mean scores on usefulness of training and resources post-training and at 3-month follow-up

	Post-training (out of 4)	Follow-up (out of 4)
*Usefulness of the training in addressing gaps in*:		
Knowledge	3.9	
Skills	3.8	
Confidence	3.8	
*Usefulness of each of the training resources*		
Video clips	3.5	
Guidance on recording smoking information in eMaternity	3.9	
Information on NSW Quitline	3.4	
Comparison with action for other conditions	3.6	
*Usefulness of the resources in working with women*		
Summary guide of 5As	4	3.3
Assist and arrange follow-up flip booklet	3.6	3.3
Helpful hints for clinicians	3.7	3.0
Reference card to pin on badge holder	3.6	3.3
Self-help booklet for use with women	3.9	3.3
NRT information sheet for clinicians	3.8	3.5
NRT information for women	3.8	3.8
*Ease of using MOHMQuit processes and resources in practice*	3.5	3.5
*Overall, how do you feel about MOHMQuit?*	3.9	3.3

Scores on the *Capability*, *Personal Priority* and *Work Environment* factors from the surveys with midwives prior to training, immediately post-training and at 3-month follow-up are shown in Table 4. There was an increase in all scores, most markedly on the *Capability* factor. While the increase on the *Personal Priority* factor was less marked, it had a high baseline and exhibited ceiling effects (maximum score of 15).

**Table 4:** Median scores on barrier and enabler factors from midwife surveys

Factor	Pre-training	Post-training	3-month follow-up
Capability (max 60)	38.5	55.0	51.0
Personal priority (max 15)	12.0	15.0	13.5
Work environment (max 25)^a^	16.0		17.5

^a^Items related to work environment were not included immediately post-training as these would not have changed

There was only one suggestion for improving the survey instruments (related to response options for model of care), with participants indicating the instruments were easy to understand and complete, and all took less than 10 min.

Despite these positive findings, there were a number of problems identified, both through the interviews with leaders and our own observations and reflections. Many of these were minor logistical issues and are not presented here. Below, we present important concerns related to the design and delivery of the MOHMQuit intervention.

##### Leadership

While the leaders participated enthusiastically in the training and continued to be enthusiastic about MOHMQuit at the follow-up interviews, they did not undertake many of the leadership roles and activities that were intended. This included repeatedly delaying the midwives’ training and only inviting a limited number of midwives, rather than all midwives providing antenatal care. Following the midwives’ training, they reported not providing an ongoing discussion of SCS or reminding midwives of its importance. However, one of the clinical educators who had missed the leadership training but attended the midwife training had provided some additional training (using MOHMQuit resources) for midwives who had missed the initial training and intended to continue providing training as part of her training schedule. The interviews revealed considerable variation in the understanding of ‘champions’, but none of the leaders had provided any support or guidance to developing them, beyond supporting midwives to attend the training. Those in management roles reported that there were many conflicting priorities and that they were time poor and thus had not been as involved as they would like to have been. However, they had observed the enthusiasm among the midwives and believed the midwives would carry the programme forward, even without the intended leadership.

##### Use of tools and resources

One of the leadership groups ran the *e*Maternity report on provision of SCS in their clinic, but had not used it for discussions with team members prior to our follow-up interview. She reported that she had intended to run it monthly and had ideas for how to use the information, but this had not yet eventuated. None of the leadership groups had completed the action planning tool or developed any local care pathways. Although the leaders all indicated in their interviews that they valued the resources for midwives, they had not been proactive in supporting their use. There was some confusion among midwives and also the clinic coordinator about whether the resources had received the appropriate approvals for use from the maternity service executive, and consequently, the resources had not been implemented.

#### Subsequent revisions to MOHMQuit

The MOHMQuit intervention is being trialled in eight maternity services across NSW. Based on the findings and experience from the feasibility study, a number of additional components are being added prior to the trial, specifically to support leaders in their role (enablement). These include engagement with CMCs in each participating site prior to commencement, to clarify expectations regarding their roles in supporting practice change and implementation of evidence-based care as they have a critical role in strategic development and service delivery and can support the clinic leaders in their role; development of a ‘community of practice’ [35] once sites have received their initial training; greater clarity in the leadership training regarding expectations and next steps, with support to commence the action planning; and assistance for leaders in developing a ‘roadmap’ to plan next steps and deadlines for actions. This final version of the MOHMQuit intervention is specified in Additional file 4 using the TIDieR checklist.

## Discussion

This paper describes a systematic, theory-informed process to develop an intervention to improve provision of guideline-recommended SCS to pregnant women who smoke. The MOHMQuit intervention takes a whole of system approach, with elements for all clinicians involved in antenatal care in the public health system (obstetricians, midwives and Aboriginal health workers), and for maternity service leaders. For each clinician group, there is training specifically addressing gaps in skills and confidence, and self-help resources to use with women. At the broader system level, the intervention includes environmental restructuring (e.g. provision of reminders, the ‘Assist and arrange follow-up flip booklet’ and ‘helpful hints for clinicians’, modifications to the EMR) and multiple mechanisms to support leadership for smoking cessation. The intervention was developed using the BCW and TDF [28, 29], and the process was consistent with recommendations for developing complex interventions, including being attentive to future implementation in real-world settings and designing and refining interventions using iterative cycles with stakeholder input throughout the process [36]. MOHMQuit uses multiple intervention types—education, training, enablement, environmental restructuring, persuasion, incentivisation and modelling, with the aim of achieving sustained culture change within the antenatal service.

Although we did not initially intend to undertake change at the system level, the use of the BCW/TDF method resulted in the identification of system-level barriers to provision of consistent SCS, including problems with the EMR, lack of quality self-help resources, no mechanisms for self-monitoring and inadequate leadership. These issues were only made explicit because we specifically asked questions about the TDF domains of behavioural regulation, environmental context and resources, and social influences. The use of the TDF to inform our primary data collection (both the interviews and the survey) ensured that we intentionally covered all TDF domains from the beginning. While the BCW method can be applied to pre-existing literature and data, particularly if resources are limited, this approach risks incomplete coverage of critical issues, and retrospective mapping to the TDF domains and BCW categories may be challenging [37].

The use of the TDF and BCW method with mapping to BCTs was particularly useful in identifying additional options which we may not have otherwise considered, including ‘action planning’, ‘self-monitoring’ and ‘comparative imagining of future outcomes’. In our feasibility study, the process of comparing care pathways and interventions for gestational diabetes, with those for smoking, was particularly powerful in engaging both the leaders and the midwives in recognising gaps in care. The demonstration of the *e*Maternity reports confirming current poor provision of SCS in their service reinforced this response.

Conducting the feasibility study was critical as it confirmed that the midwifery component was well-targeted, acceptable and feasible, with minimal changes required prior to our larger trial. There were increases on the *Capability*, *Personal Priority* and *Work Environment* factor scores, particularly the *Capability* factor. The *Personal Priority* scores were initially high and exhibited ceiling effects. However, the leaders indicated that they had intentionally invited midwives who were known to be more interested in addressing smoking, which may account for the high baseline scores. The request for ongoing/follow-up training from some midwives confirmed the value of the train-the-trainer approach, but also reinforced the need to ensure the clinical midwifery educators engage with the intervention from the start.

The feasibility study also identified the need for further strengthening of the leadership component. Although our initial data collection identified the need for improved leadership, it did not fully explore the barriers to provision of this leadership. For this, the project would have been strengthened by an additional study, specifically exploring these issues. The additional enablement components added following the feasibility study were developed through discussions within the research team (including CA, a senior midwife), with consideration of the context, and usual processes used within the health system. These may be further refined through discussion with CMCs and other senior midwives at sites participating in our larger trial, prior to its commencement.

We had strong engagement with key stakeholders from project inception. The project was initiated following discussions with a senior midwife (CA) who was a core member of the research team throughout. Her role within the health system also facilitated engagement with senior officials in NSW Health, and with other maternity service providers across the state. Through the Advisory Group, meetings with key policymakers at the Ministry of Health, and the stakeholder workshop, we were able to ensure that the intervention developed was suitable to the context, consistent with NSW Health priorities and policies, and acceptable to core target groups. Others have recognised that despite the highly structured approach of the BCW method, it is not a rigid process but requires the team to make subjective and pragmatic decisions throughout [38]. We see this as a strength, as it allows for flexibility and adaptation to the specific context and the particular issue of concern. In our case, the involvement of stakeholders at multiple levels of the system and in every step of the process meant that the researchers were not making decisions in isolation, but were strongly guided by these groups and able to ensure the intervention was suited to the context.

Involvement of stakeholders in several branches of the Ministry of Health (Nursing and Midwifery, Obstetrics, Tobacco Control, and Epidemiology and Evidence), the Cancer Institute NSW, and multiple LHDs also ensured general awareness of the project across the health system and willingness to engage with the research team in multiple ways. This engagement enabled the rapid translation of some critical findings to improve the development of the new EMR and use of the findings in the development of online training modules. These stakeholders have now partnered with us for the larger cluster-randomised stepped-wedge trial of the MOHMQuit intervention, funded by the National Health and Medical Research Council. Despite these positive outcomes from the stakeholder engagement, the timeframe required for the full process made this engagement challenging to maintain, and there was some turnover among organisational representatives.

Sustainability was a critical consideration in the development of the MOHMQuit intervention. For example, in developing an audit and feedback process, rather than having this dependent on the research team, we deliberately created a mechanism for the clinic leaders to undertake the process through a reporting template in *e*Maternity, with guidance on how to use this for quality improvement. Other examples of designing for sustainability include the service action planning tool, guidance on developing champions, guidance on developing clinical pathways, and the train-the-trainer model, all of which support local leadership in implementing MOHMQuit and enable ongoing refinement. In addition to supporting sustainability, these tools support adaptation of the intervention to suit the local context, an important consideration when designing interventions for broad implementation [39]. It will be critical to assess the uptake of these elements, how they are used, and longer term sustainability of the MOHMQuit intervention as part of the process evaluation of the implementation trial.

Several other groups have used the COM-B or TDF to develop implementation interventions with demonstrated success in improving SCS. The BabyClear intervention implemented in North East England was developed based on findings from a study using the TDF [40] and demonstrated a doubling of referrals to quit smoking services and a significant increase in quitting by the time of delivery [41]. Gould and colleagues used the BCW method to design the Indigenous Counselling and Nicotine (ICAN) QUIT intervention for delivery in Australian Aboriginal Medical Services [42] with good acceptability and feasibility demonstrated in a small pilot study [43]. Informed by the Consolidated Framework for Implementation Research [44], researchers in the Netherlands developed online information sheets and text messages, tailored to different health professional groups (gynaecologists, midwives, paediatricians, practice nurses and respiratory nurses) with demonstrated feasibility, acceptability and reach [45]. While there were significant improvements in both tested and self-reported knowledge among all groups, only paediatricians increased provision of quit advice [45]. However, the low cost of this intervention makes it highly scalable and further research assessing its sustainability is warranted. Similar digital approaches may also be valuable in sustaining the impact of interventions such as MOHMQuit. For all these interventions, the theoretical underpinnings, and detailed process of design need to be well described, to support understanding of what works, for whom and in what circumstances, and thus advance implementation science in this area.

### Strengths and weaknesses

Important strengths of this project have been discussed above, including explicit use of theory, strong engagement with key stakeholders from inception, and the feasibility study with subsequent refinements. The clear specification of the intervention development using this process—i.e. identifying barriers to SCS and specifying specific intervention types and behaviour change techniques aimed at addressing them, also supports the design of the evaluation to assess implementation fidelity, testing the intended mechanisms of action and future replication. The most critical weakness was that we did not explicitly explore the barriers and enablers to providing SCS leadership within maternity services. Utilising an additional theoretical framework to assess the organisational context of antenatal care, such as the Model of Diffusion of Innovations in Service Organisations [46], would likely have assisted to address this and other organisational barriers to change. Doing this would have enabled us to include more specifically targeted intervention types and BCTs to address these barriers.

## Conclusions

Using the BCW method combined with strong stakeholder engagement from inception has resulted in transparent development of the MOHMQuit intervention, which targets identified barriers to provision of SCS and is developed specifically for the context in which it will be implemented. While others have developed training programmes for antenatal clinicians to improve their provision of SCS, we are not aware of any that have explicitly addressed leadership for SCS nor the broader systemic issues such as the capability of the EMR to support clinicians in this role. The MOHMQuit intervention is being trialled in eight maternity services in NSW, using a pragmatic cluster-randomised stepped-wedge design. The trial includes multiple outcome measures including changes in clinician and leader behaviour; changes in women’s smoking behaviour; a process evaluation to assess acceptability, adoption, appropriateness, feasibility, fidelity, penetration, sustainability and reach and to test the mechanisms of action; and a cost-effectiveness analysis. The stepped-wedge design has the advantage of being able to assess when changes occurred relative to the provision of the intervention, the possible impact of external factors such as policy changes and the extent to which changes are sustained.

## Supplementary Information

**Additional file 1.** Provide further details regarding the barriers and enablers to provision of smoking cessation support identified in our initial research.

**Additional file 2.** Shows the results of Step 5 – Use of the APEASE criteria to identify potentially relevant intervention functions.

**Additional file 3.** Shows the results of Step 7 – Use of the APEASE criteria by the stakeholder workshop participants to review potential BCTs.

**Additional file 4.** Completed TIDieR checklist for the MOHMQuit intervention.

## Data Availability

All the materials are available in the additional files.

## Abbreviations

5As: Ask, Advise, Assess, Assist, Arrange follow-up
APEASE: Affordability, Practicability, Effectiveness and cost-effectiveness, Acceptability, Side effects/safety, Equity
BCT: Behaviour Change Technique
BCW: Behaviour Change Wheel
CMC: Clinical Midwifery Consultant
COM-B: Capability, Opportunity, Motivation - Behaviour
EMR: Electronic medical record
HETI: Health Education and Training Institute
LHD: Local Health District
MoH: Ministry of Health
MOHMQuit: Midwives and Obstetricians Helping Mothers to Quit
NRT: Nicotine replacement therapy
NSW: New South Wales
SCS: Smoking cessation support
TDF: Theoretical Domains Framework
