# Supplementary material for: Improving implementation of smoking cessation guidelines in pregnancy care: development of an intervention to address system, maternity service leader and clinician factors
Source: Implement Sci Commun. 2021 Nov 17;2:128. doi: 10.1186/s43058-021-00235-5 (PMC8597300; doi:10.1186/s43058-021-00235-5)
Supplement: Supplementary file 1 — Additional file 1. Provide further details regarding the barriers and enablers to provision of smoking cessation support identified in our initial research. [file 43058_2021_235_MOESM1_ESM.docx]

**Additional File 1.**

**Further detail on the barriers and enablers to provision of Smoking Cessation Support**

Psychological capability

While knowledge of the harms of smoking was reasonably strong, many clinicians were not aware of the guidelines or the 5As and there was confusion regarding the benefits of quitting smoking versus cutting down(1, 2). Clinicians also reported poor knowledge and skills related to assisting women to quit smoking and few had any systems for monitoring their own provision of cessation support(1, 2). However, many also recognised that midwives generally have good communication skills and were a trusted source of information for women, which was a potential enabler for clinicians’ skill development specific to SCS(1).

Physical opportunity

Several factors relating to physical opportunity impacted clinicians’ provision of SCS, including lack of systems to identify smokers or to monitor provision of SCS within the clinic(1, 2). While the EMR in use at the time included a mandatory prompt to record smoking status at the initial visit, at subsequent visits there were no prompts and it was difficult to identify both smoking status and what support women had been offered. There were no identified fields for recording smoking-related information at subsequent visits, which resulted in information being recorded in multiple different sections, or not at all. A further consequence of this problem was that it was not possible for managers or others to monitor SCS provision. Other issues impeding provision of SCS were a perception that providing SCS took too long in a busy antenatal visit, and a there was a lack of pregnancy-specific cessation resources to use with women(1, 2).

Social opportunity

While some clinicians reported that there were local smoking cessation champions, this was rare and there was a lack of leadership for SCS among both managers and peers, with the perception that SCS was not a service priority(1, 2).

Reflective motivation

Multiple reflective motivation factors impacted provision of SCS, including lack of confidence in assisting women, especially if the woman was struggling to quit; a perception that referral to the state Quitline was not effective; and concerns that women did not want to discuss their smoking and that doing so would damage the relationship midwives had with women(1, 2). There was also a perception among some midwives that smoking was a social issue or ‘lifestyle choice’ and therefore not a core aspect of a clinical consultation. In these circumstances, conditions considered more medical (e.g. gestational diabetes) were prioritised(1). Importantly, other midwives reported increased role satisfaction in helping women addressing their smoking(1, 2).

Automatic motivation

Some midwives were uncomfortable discussing smoking with women(1, 2), which was in part due to their lack of confidence, knowledge and skills regarding providing SCS and also due to the perception that smoking was a ‘lifestyle choice’(1).

1. Longman JM, Adams CM, Johnston JJ, Passey ME. Improving implementation of the smoking cessation guidelines with pregnant women: what might help clinicians? Midwifery. 2018;58:137-44.

2. Passey ME, Longman JM, Adams C, Johnston JJ, Simms J, Rolfe M. Factors associated with provision of smoking cessation support to pregnant women - a cross-sectional survey of midwives in New South Wales, Australia. BMC Pregnancy and Childbirth. 2020;20:219.
