# Supplementary material for: Improving implementation of smoking cessation guidelines in pregnancy care: development of an intervention to address system, maternity service leader and clinician factors
Source: Implement Sci Commun. 2021 Nov 17;2:128. doi: 10.1186/s43058-021-00235-5 (PMC8597300; doi:10.1186/s43058-021-00235-5)
Supplement: Supplementary file 2 — Additional file 2. Shows the results of Step 5 – Use of the APEASE criteria to identify potentially relevant intervention functions. [file 43058_2021_235_MOESM2_ESM.docx]

**Additional File 2.**

**Step 5. Use of APEASE criteria to identify potentially relevant intervention functions**

| **Intervention Functions** | **Affordability** | **Practicability** | **Effectiveness & & cost effectiveness** | **Acceptability** | **Side effects/**  **safety** | **Equity** | **Comments** | **Decision:**  **Yes/No for next phase** |
| --- | --- | --- | --- | --- | --- | --- | --- | --- |
| Education | ✓ | ✓ | ✓ | ✓ | ✓ | ✓ | Providing information (online or face to face) to increase midwives’ knowledge of the 5As, the NSW Health policies and guidelines, the risks/benefits of quitting vs cutting down, the effectiveness of Quitline and smoking as an addiction is affordable, practicable, effective, acceptable and could be equitable if easily accessible e.g. online. NSW Health has a policy of providing education/on-going CPD to clinical staff, and has previously provided face to face training for priority issues such as smoking cessation. We do not anticipate any unwanted consequences. | Yes |
| Training | ✓ | ? | ✓ | ✓ | ✓ | ✓ | Providing training to develop required skills through face to face workshops is affordable, effective, acceptable to midwives and can be addressed equitably (using a ‘road show’ model of delivery). Within NSW the model for training is moving more towards online, so will clinicians have time and support to attend? Many of the components are not feasible using online training e.g. buddying, practising and feedback. We do not anticipate any unwanted consequences. | Yes |
| Enablement (a) | ✓ | ? | ? | ✓ | ✓ | ✓ | Buddying midwives for on-going support between and after training is affordable, acceptable and equitable, and we do not anticipate any unwanted consequences. However, there are questions over the practicability and effectiveness of buddying – will midwives embrace this idea and do it? Will rosters and other workplace issues support it? | Yes |
| Enablement (b) | ✓ | ✓ | ✓ | ✓ | ✓ | ? | Brainstorming/action planning/problem solving at the individual midwife level; and restructuring the social environment by working with managers (in a separate process) to encourage them to support training, monitor performance of service, add 5As to team meeting discussions etc. These intervention functions are affordable, practicable, effective and acceptable and we do not anticipate any unwanted consequences. However, there may be issues around equity given the very different management structures of different services | Yes |
| Environmental Restructuring (a) | ? | ? | ✓ | ? | ✓ | ✓ | The new EMR (*e*Maternity) now flags smokers, so identifies them at subsequent visits. It doesn’t remind them to follow 5As. Can record smoking cessation support clients have received, and this can be found if sought at subsequent visits. However, system doesn’t provide any support to assist the midwife to provide Advice or Assistance. Further amendments to *e*Maternity are not feasible at this stage but may be in future. Option of developing a linked add-on electronic decision support system is highly unlikely to be acceptable to MoH and is probably not affordable. Likely to be very effective.  So, alternative options would include: paper-based reminders to follow 5As. Prompts/cues; poster on wall; prominent list of what to do (colourful and attractive). These are affordable, practicable, acceptable, equitable and unlikely to have unwanted consequences. Effectiveness unknown. | Yes, definitely need expert input |
| Environmental Restructuring (b) | ✓ | ✓ | ? | ✓ | ✓ | ✓ | Resources – making available up-to-date self-help materials brochures and other visual resources for midwives to use with women. These are affordable, practicable, acceptable, equitable and unlikely to have unwanted consequences. Not effective if used in isolation, but may be effective as part of a complex intervention and when used as a prompt for discussions. | Yes |
| Persuasion | ✓ | ✓ | ? | ✓ | ? | ✓ | Providing video clips (patients, midwives, about the Quitline), a focus on past successes, all from credible sources. Information on health consequences that compare smoking with other pregnancy complications and reframing smoking as an addiction, not a lifestyle choice. These intervention functions are affordable, practicable, acceptable and equitable. However, there is a question around effectiveness and it is possible that the video clips may generate unwanted consequences by stimulating cynicism. | Yes |
| Incentivisation | ✓ | ✓ | ? | ✓ | ✓ | ✓ | Social rewards – praise for practising behaviour during and between the training sessions  This intervention function is affordable, practicable, acceptable, equitable and we do not anticipate any unwanted consequences. We are unsure about its potential effectiveness. | Yes |
| Modelling | ✓ | ✓ | ✓ | x | x | ✓ | Providing a video of an engaged client and an effective midwife is affordable, practicable (more practicable than face to face demonstration), effective, and equitable in relation to access. However, there is potential for negative reaction and perception that the video scenario is not realistic which may limit acceptability and generate unwanted consequences | Yes |
| Restriction | - | x | x | x | - | - | This intervention function concerns using rules to increase the target behaviour by reducing the opportunity to engage in competing behaviours. As it is inappropriate to restrict competing behaviours then this is not appropriate. | No |
| Coercion | - | x | ? | x | x | - | Create an expectation of punishment or cost. This would be entirely unacceptable to midwives and it is not practicable in terms of defining suitable punishments or costs or a system to ensure punishments were implemented within constrained resources of the health system. Not appropriate. | No |

**Key**: ✓ = Yes; x = No; ? = Unsure; - = not assessed
