# Supplementary material for: Improving implementation of smoking cessation guidelines in pregnancy care: development of an intervention to address system, maternity service leader and clinician factors
Source: Implement Sci Commun. 2021 Nov 17;2:128. doi: 10.1186/s43058-021-00235-5 (PMC8597300; doi:10.1186/s43058-021-00235-5)
Supplement: Supplementary file 3 — Additional file 3. Shows the results of Step 7 – Use of the APEASE criteria by the stakeholder workshop participants to review potential BCTs. [file 43058_2021_235_MOESM3_ESM.docx]

**Additional File 3.**

**Step 7. Use of APEASE criteria by the stakeholder workshop to review potential BCTs**

| Suggested BCTs | **A**ffordability | **P**racticability | Likely **E**ffectiveness | **A**cceptability | **S**ide effects | **E**quity | Keep? | Critical?  (1-10) | Comments |
| --- | --- | --- | --- | --- | --- | --- | --- | --- | --- |
| **Education** | | | | | | | | | |
| *6.3 Information about others approval, 9.1* *Credible source*. Providing information from credible sources about policy and guidelines to increase midwives’/leaders’ knowledge of the 5As. (*midwives & leaders**) | ✓ | ✓ | ✓ | ✓ | ✓ | ✓ | ✓ | 10 | Acceptability need to be aware of possible push-back that may affect acceptability. Side effects may include feeling overwhelmed, due to many priorities and resultant fatigue |
| *4.1 Instruction on how to perform a behaviour, 9.1* *Credible source.* Provide information from credible sources on what is involved in providing each of the 5As and when they should be provided. (*midwives & leaders*) | ✓ | ✓ | ✓ | ✓ | ? | ✓ | ✓ | 10 | Practicability – Possibly build into an online module – but will require face to face too. Equity - If not blended with face to face training then somehow need to have time and safety to practice the skills in a learning environment. Costs and effectiveness – mostly associated with midwives’ time for training. Unwanted side effects – consider ‘training fatigue’, a cumulative risk to enthusiasm. |
| **Training** | | | | | | | | | |
| *4.1 Instruction on how to perform a behaviour, 6.1 Demonstration of the behaviour, 8.1 Behavioural practice/rehearsal.* Demonstration (video) of how to motivate, plus role play and feedback. *(midwives only)* | ✓ | ✓ | ? | ✓ | ✓ | ✓ | ✓ | 9 | Effectiveness – Videos, assuming they are done very well, are great training devices, but if the only resource then won’t be effective. They need to be part of a suite of resources. |
| *4.1 Instruction on how to perform a behaviour, 6.1 Demonstration of the behaviour, 8.1 Behavioural practice/rehearsal.* Demonstration (video) of how to provide assistance (including NRT) and follow up, plus role play and feedback. *(midwives only)* | ✓ | ✓ | ? | ✓ | ✓ | ✓ | ✓ | 9 | Practicability – If the videos are short enough they can be spliced into other opportunities like hand-over, in-service etc. |
| *4.1 Instruction on how to perform a behaviour, 6.1 Demonstration of the behaviour, 8.1 Behavioural practice/rehearsal.* Demonstration (video) and practise of how integrate following the 5As into routine clinical skills e.g. taking a blood pressure. *(midwives only)* | ✓ | ✓ | ? | ✓ | ✓ | ✓ | ✓ | 9 | Acceptability and Effectiveness – Concerns that midwives don’t like doing role play and this may impact both acceptability and effectiveness. |
| *4.1 Instruction on how to perform a behaviour.* Instruction on HOW to: provide leadership in smoking cessation support; identify enthusiasts and turn them into champions; use resources, prompts, cues to greatest effect; run reports from eMaternity. (*leaders only*) | ✓ | ✓ | ? | ✓ | ? | ? | ✓ | 5 | Effectiveness – Questions about whether leaders will take these opportunities up. Equity – rural leaders getting access to any face to face training; and non-*e*Maternity users may be disadvantaged. |
| **Enablement** | | | | | | | | | |
| *3.1 Social support (unspecified).* A buddy identified at the first training to provide on-going support. *(midwives only)* | ✓ | X | X | ✓ | ✓ | ✓ | ? | 5 | Practicability – Issues with this mainly because of logistics (shift work, lack of continuity, and the rural/urban dimension etc.) Effectiveness is entirely dependent on logistics, how good the buddy is, and how much support and training the buddy gets both beforehand and during the buddying. |
| *1.4 Action planning, 1.2 Problem solving, 2.2 Feedback on behaviour, 2.3 Self-monitoring.* Getting midwives to brainstorm and action plan ways for them to monitor how well they are following the 5As. *(midwives only)* | ✓ | ✓ | ✓ | ✓ | ✓ | ✓ | ✓ | 8 | Acceptability – Liked this idea as it is strengths-based, calling on existing knowledge and valuing what midwives already know. Practicability – Needs to be a simple, clear process with good guidelines. Requires support from leaders to do this. |
| *12.2 Restructuring the social environment.* The leaders encouraging attendance at training. (*leaders only*) | ✓ | ✓ | ? | ✓ | ✓ | ✓ | ✓ | 8 | Effectiveness relates to leaders’ own beliefs and competing interests. High interpersonal variability will have a major impact on effectiveness. Proactive leaders are necessary – their competing priorities are an issue so need endorsement from more senior leadership. Training quality is crucial to ongoing endorsement. Practicability depends on training timetable. Seen as fundamental and very important. |
| *12.2 Restructuring the social environment.* The leaders encouraging discussion of 5As in team meetings. (*leaders only*) | ✓ | ✓ | ✓ | ✓ | ✓ | ✓ | ✓ | 8 | Effectiveness depends on the leader’s skills at the 5As. More likely to be effective in small local environment where honest discussions of skills and practical tips can be shared – important to do. Practicability - there is already too much to cover in existing agenda. Alternative is to do it as part of a ‘blitz’ or short spurts throughout the year e.g. *5As in August* |
| *2.2 Feedback on behaviour, 12.2 Restructuring the social environment.* Developing a mechanism to allow the leaders to monitor and communicate progress on the 5As and discussing this at team meetings/display in staff tea room etc. (*leaders only*) | ✓ | ✓ | ✓ | ✓ | ✓ | ✓ | ✓ | 8 | Practicability – Depends on the mechanism, if quick and easy ‘click’ then it will work. Could encourage peer comparison to produce desire to improve and look at how others do better/well. Possible side-effect – could get push-back if it becomes part of performance criteria for individuals. Will be accepted if it is built into the system rather than being an add-on. Proper reporting against a KPI is needed. Seen as fundamental and very important – essential to ongoing impact. |
| *2.2 Feedback on behaviour, 1.4 Action planning, 1.2 Problem solving.* Getting leaders to brainstorm and action plan how to improve implementation of the guidelines in their service. (*leaders only*) | ✓ | ✓ | ✓ | ? | ✓ | ? | ✓ | 7 | Consider equity for those who need to travel – mode of meeting is important. Acceptability - need top-down support. Effectiveness – Leader skills and passion important. |
| **Environmental restructuring** | | | | | | | | | |
| *7.1 Prompts/cues, 12.5 Adding objects to the environment*. Modify *e*Maternity (the EMR) to include flags for smokers (now complete) and build in reminders to follow 5As at every antenatal visit. *(system wide)* | ✓ | x | ✓ | ✓ | ✓ | x | ✓ | 10 | Seen as essential. Affordable because Ministry has committed to do it – it’s going ahead anyway and will continue to be developed in an evolutionary manner. Practicability – not practicable for midwives not sitting in front of a computer when providing care, and some LHDs not having *e*Maternity. Training in use will be critical. Acceptability - if it helps, midwives will accept it, but not if it’s difficult to use/not helpful. Equity – non-clinical setting, no computers. 2 LHDs don’t use *e*Maternity. |
| *12.5 Adding objects to the environment*. Develop a reporting system for leaders to monitor cessation support provided. *(system wide)* | ✓ | ✓ | ✓ | ✓ | ✓ | ✓ | ✓ | 9 | Seen as essential. Ministry is committed to improving *e*Maternity, so this would be an excellent addition. Considered practicable, acceptable, equitable and likely to be effective but this will depend on the leader. No unwanted consequences perceived |
| *7.1 Prompts/cues, 12.5 Adding objects to the environment*. Developing a linked add-on electronic decision support system. *(system wide)* | x | x | - | x | ? | x | x | - | Practicability – Would require permission from *e*Maternity, which may not be forthcoming. Challenges with need to keep updated, real question of sustainability and governance if not part of central system. Effectiveness – likely to be effective where its installed, if well done. Equity – not everyone would opt-in. Should test the feasibility of this if changes to *e*Maternity stall or are not possible. |
| *7.1 Prompts/cues, 12.5 Adding objects to the environment*. Paper-based reminders to follow 5As (If above not feasible). *(system wide)* | ✓ | x | ? | x | - | - | x | - | Affordability – in the short-term. Practicability - Two options discussed – a checklist type that doesn’t involve any recording of information - not good for continuity of care as can’t tell what has happened previously. Record-keeping/care plan model (similar to versions currently in use in some LHDs) – too onerous and risks duplicating recording of information etc. Might work to prompt but not work if used as a care plan. Acceptability - Paper-based systems seen as going backwards! Side Effects - Keeping them updated is problematic |
| *7.1 Prompts/cues, 12.5 Adding objects to the environment*. Prompts/cues; poster on wall; prominent list of what to do (colourful and attractive). *(system wide)* | ✓ | ? | ? | ✓ | ✓ | ✓ | ✓ | 4 | Practicability – Having them available is important, but not essential and don’t make them compulsory as clinics vary. |
| *12.5 Adding objects to the environment.* Central (up to date/updated) resources (for women) linked out from *e*Maternity on: Motivating quit attempts; Strategies for quitting; Using NRT; Postpartum relapse prevention etc. *(system wide)* | ✓ | ✓ | ✓ | ✓ | ✓ | ✓ | ✓ | 10 | This should be included. Affordability – yes as there is currently momentum and this is more efficient than everyone developing their own. Practicability - All in one place and maintained as current so very practicable and acceptable. Acceptability – yes for reasons above, but some disagreement about whether women want brochures, so consider multiple forms of resources – brochures, apps etc. Centralising may not be acceptable to those running eMaternity, so also consider other sources (eg print, web-based) |
| **Persuasion** | | | | | | | | | |
| *9.1 Credible source, 15.3 Focus on past success, 15.1 Verbal persuasion about capability.* Using a credible source to highlight the communication skills midwives have developed in other areas. *(midwives only)* | ✓ | ✓ | ✓ | ✓ | ✓ | ✓ | ✓ | 7 | Effectiveness – need practice and support opportunities/mentoring for new/junior staff/students. |
| *9.1 Credible source, 5.1 Information about health consequences.* Using a credible source to provide information that referrals to Quitline (or other referral service) are effective. (*midwives & leaders*) | ✓ | ✓ | ? | ✓ | ✓ | ? | ✓ | 6 | Keep IF it is part of other options. Need to raise profile and understanding of Quitline including effectiveness. Issues (equity, effectiveness, side effects) re disadvantage associated with Quitline – women change phones, don’t have credit to call. |
| *5.1 Information about health consequences, 5.2 Salience of health consequences, 9.3 Comparative imagining of future outcomes, 13.2 Framing/reframing.*  Use of a comparison e.g. gestational diabetes to reframe smoking as a critical clinical issue and complication of pregnancy that should be addressed. (*midwives & leaders*) | ✓ | ✓ | ✓ | ✓ | X | ✓ | ✓ | 8 | Side effects – Wary of it being classified as a risk and women being classified as ‘high risk’ (if they are classified as high risk they won’t get access to the MGP which is what they need for cessation. |
| *5.1 Information about health consequences, 9.1 Credible source, 9.3 Comparative imagining of future outcomes, 13.2 Framing/reframing.* Reframing smoking as an addiction rather than a social/lifestyle choice – providing information from a credible source about the role of nicotine in addiction. (*midwives & leaders*) | ✓ | ✓ | ✓ | ✓ | ✓ | ✓ | ✓ | 10 | It is important and can stand on its own. A bit hard to give broad assessment of effectiveness given individual differences/attitudes. Also need to consider turn-over of staff and ensuring new midwives receive education. Having a true understanding of nicotine addiction will give midwives the compassion and understanding of how difficult quitting can be for some women. |
| *9.1 Credible source, 5.4 Information about social and environmental consequences.* Video clip of an expert patient describing health and emotional consequences (not feeling valued) of midwife NOT addressing their smoking.  *(midwives only)* | ✓ | ✓ | ? | ✓ | ✓ | ✓ | ✓ | 7 |  |
| **Incentivisation** | | | | | | | | | |
| *10.4 Social reward* Praise for practising behaviour during and between intervention training sessions (from buddy or local champion). *(midwives only)* | ✓ | ✓ | ? | ✓ | ✓ | ✓ | ✓ | 7 | Practicability - Buddy may not be practical, but this role is important and could be provided by the CME or other champion. |
| **Modelling** | | | | | | | | | |
| *4.1 Instruction on how to perform a behaviour, 6.1 Demonstration of the behaviour.* Demonstration of the behaviour - video showing midwife offering smoking cessation support without damaging the client relationship. *(midwives only)* | ✓ | ✓ | ? | ✓ | ? | ✓ | ✓ | 4 | Can help as don’t always see other midwives doing things so can provide a light-bulb moment “wow that’s how you do it!” Effective if done the right way (not too long) and combined with practice and feedback (blended learning, face to face, online). Side effects – may be seen as not real/not credible/not “my clinical situation”. Acceptability – Easy, accessible, social and timely. |

* The term leaders refers to midwifery managers, educators and those in other leadership roles

**Key**: ✓ = Yes; x = No; ? = Unsure; - = not assessed
