# Supplementary material for: Improving implementation of smoking cessation guidelines in pregnancy care: development of an intervention to address system, maternity service leader and clinician factors
Source: Implement Sci Commun. 2021 Nov 17;2:128. doi: 10.1186/s43058-021-00235-5 (PMC8597300; doi:10.1186/s43058-021-00235-5)
Supplement: Supplementary file 4 — Additional file 4. Completed TIDieR checklist for the MOHMQuit intervention. [file 43058_2021_235_MOESM4_ESM.docx]

**Additional file 4 – Completed TIDieR checklist**

**
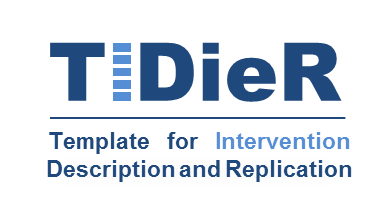
The TIDieR (Template for Intervention Description and Replication) Checklist*:**

Information to include when describing an intervention and the location of the information

| Item number | Item |  |
| --- | --- | --- |
|  | **BRIEF NAME** |  |
| **1.** | Provide the name or a phrase that describes the intervention. | Midwives and Obstetricians Helping Mothers to Quit (MOHMQuit) intervention |
|  | **WHY** |  |
| **2.** | Describe any rationale, theory, or goal of the elements essential to the intervention. | The MOHMQuit intervention was developed using the Behaviour Change Wheel method in order to improve implementation of guidelines for supporting smoking cessation during pregnancy, among antenatal care providers working in the public health system. |
|  | **WHAT** |  |
| **3.** | Materials: Describe any physical or informational materials used in the intervention, including those provided to participants or used in intervention delivery or in training of intervention providers. Provide information on where the materials can be accessed (e.g. online appendix, URL). | The MOHMQuit intervention includes the following materials:  For leaders (midwifery unit managers, clinical midwifery consultants, clinical midwifery educators, other senior midwives):   - Template for generating *e*Maternity reports on provision of SCS in their clinic - Guidance on use of the *e*Maternity reports for quality improvement - Comparison with action for other conditions e.g gestational diabetes - A clinic/service action planning tool - Guidance on developing champions - Guidance on developing local care pathways   For clinicians (midwives, Aboriginal Health Workers and obstetricians):   - 11 short videos demonstrating critical techniques in providing SCS - Guidance on recording smoking information in *e*Maternity - Information on NSW Quitline - Comparison with action for other conditions e.g gestational diabetes - Summary guide of the 5As - Assist and arrange follow-up flip booklet - Helpful hints for clinicians - Reference card to pin on badge holder - Self-help booklet for use with women - NRT information sheets for clinicians - NRT information for women |
| **4.** | Procedures: Describe each of the procedures, activities, and/or processes used in the intervention, including any enabling or support activities. | The MOHMQuit intervention includes several stages:  All participants will be requested to complete two short online training modules developed by the NSW Health Education and Training Institute (HETI), prior to other components. These modules address:   - NSW Health policy regarding SCS - Knowledge of harms of smoking in pregnancy - The evidence for SCS - Use of the 5As - Use of NRT in pregnancy   Clinical Midwifery Consultants (CMCs):   - Initial engagement to clarify their role in supporting practice change and the MOHMQuit intervention   For leaders:  3-hour workshop covering:   - SCS leadership - Reviewing *e*Maternity reports (local performance data on provision of SCS) - Action planning, including initial steps - Developing care pathways for SCS - Developing and maintaining champions - Expectations regarding next steps and support for MOHMQuit within their service, including development of a plan with deadlines for actions   For midwives and Aboriginal Health Workers:  Full-day workshop covering:   - Importance of providing SCS - How to provide effective SCS - How to use the MOHMQuit resources - Using tools for self-monitoring provision of SCS and action planning - Documentation of SCS in *e*Maternity   For obstetricians and obstetric trainees:   - Importance of providing SCS - How to provide effective SCS - How to use the MOHMQuit resources - Documentation of SCS in *e*Maternity   Additionally, services will be encouraged to form a ‘Community of practice’ to provide additional and ongoing peer support and encouragement.  Clinical Midwifery Educators will be provided training and resources to continue to deliver training. |
|  | **WHO PROVIDED** |  |
| **5.** | For each category of intervention provider (e.g. psychologist, nursing assistant), describe their expertise, background and any specific training given. | Training for the leadership group will be provided by a senior CMC with extensive experience in leadership, evidence-based practice and training within NSW maternity services, and with detailed knowledge of all components of the MOHMQuit intervention.  Training for the clinical groups will be provided jointly by the CMC, and an accredited smoking cessation trainer, with experience specifically related to smoking in pregnancy. |
|  | **HOW** |  |
| **6.** | Describe the modes of delivery (e.g. face-to-face or by some other mechanism, such as internet or telephone) of the intervention and whether it was provided individually or in a group. | The HETI modules are provided on-line and It is intended that all participants will complete these in their own time before other components.  All MOHMQuit-specific training sessions are intended to be provided as face-to-face group training sessions. However, this may need to be revised in light of the COVID-19 pandemic and on-line or hybrid models will be considered, depending on circumstances.  Regardless of mode of delivery, all participants will be provided with the relevant resources for use in their services.  All clinicians (midwives, Aboriginal Health Workers, obstetricians and obstetric trainees) providing antenatal care will be asked to participate in the relevant training, and training will be offered several times at each site to maximise opportunity for participation. |
|  | **WHERE** |  |
| **7.** | Describe the type(s) of location(s) where the intervention occurred, including any necessary infrastructure or relevant features. | The MOHMQuit intervention will be provided at public maternity services in NSW. |
|  | **WHEN and HOW MUCH** |  |
| **8.** | Describe the number of times the intervention was delivered and over what period of time including the number of sessions, their schedule, and their duration, intensity or dose. | The MOHMQuit intervention will be delivered in each participating site, with one session for the leadership group and 2-3 sessions available for midwives and Aboriginal Health Workers. The training for obstetricians and trainees will be provided twice at each site. Additionally, to address the need to address staff turnover, the Clinical Midwifery Educators will be trained to provide ongoing training using a train-the-trainer model. |
|  | **TAILORING** |  |
| **9.** | If the intervention was planned to be personalised, titrated or adapted, then describe what, why, when, and how. | Several elements of the intervention can be adapted to local services including action planning and developing care pathways for SCS, which will be developed by the local services. They will also be encouraged to review these annually. |
|  | **MODIFICATIONS** |  |
| **10.^ǂ^** | If the intervention was modified during the course of the study, describe the changes (what, why, when, and how). | The intervention was modified following a small feasibility study, with additional enablement added to support the leadership group. This included engagement with CMCs in each participating site prior to commencement, to clarify expectations regarding their roles in supporting practice change and implementation of evidence-based care; development of a ‘community of practice’, once sites have received their initial training; greater clarity in the leadership training regarding expectations and next steps, with support to commence the action planning; and assistance for leaders in developing a ‘roadmap’ to plan next steps and deadlines for actions. Any subsequent changes during the larger implementation trial will be reported. |
|  | **HOW WELL** |  |
| **11.** | Planned: If intervention adherence or fidelity was assessed, describe how and by whom, and if any strategies were used to maintain or improve fidelity, describe them. | This will be assessed in the larger trial. Further details will be provided in the MOHMQuit trial protocol paper. |
| **12.^ǂ^** | Actual: If intervention adherence or fidelity was assessed, describe the extent to which the intervention was delivered as planned. | N/A. Not yet implemented. |

** **Authors** - use N/A if an item is not applicable for the intervention being described. **Reviewers** – use ‘?’ if information about the element is not reported/not sufficiently reported.

† If the information is not provided in the primary paper, give details of where this information is available. This may include locations such as a published protocol or other published papers (provide citation details) or a website (provide the URL).

ǂ If completing the TIDieR checklist for a protocol, these items are not relevant to the protocol and cannot be described until the study is complete.

* We strongly recommend using this checklist in conjunction with the TIDieR guide (see *BMJ* 2014;348:g1687) which contains an explanation and elaboration for each item.

* The focus of TIDieR is on reporting details of the intervention elements (and where relevant, comparison elements) of a study. Other elements and methodological features of studies are covered by other reporting statements and checklists and have not been duplicated as part of the TIDieR checklist. When a **randomised trial** is being reported, the TIDieR checklist should be used in conjunction with the CONSORT statement (see [www.consort-statement.org](http://www.consort-statement.org)) as an extension of **Item 5 of the CONSORT 2010 Statement.** When a **clinical trial** **protocol** is being reported, the TIDieR checklist should be used in conjunction with the SPIRIT statement as an extension of **Item 11 of the SPIRIT 2013 Statement** (see [www.spirit-statement.org](http://www.spirit-statement.org)). For alternate study designs, TIDieR can be used in conjunction with the appropriate checklist for that study design (see [www.equator-network.org](http://www.equator-network.org)).
